# Two-dimensional speckle technique for slope error measurements of weakly focusing reflective X-ray optics

**DOI:** 10.1107/S160057752200916X

**Published:** 2022-10-05

**Authors:** Lingfei Hu, Hongchang Wang, Oliver Fox, Kawal Sawhney

**Affiliations:** a Diamond Light Source Ltd, Harwell Science and Innovation Campus, Didcot, Oxfordshire OX11 0DE, United Kingdom; Tohoku University, Japan

**Keywords:** speckle technique, X-ray optics, at-wavelength metrology

## Abstract

Speckle-based at-wavelength metrology techniques now play an important role in X-ray wavefront measurements. However, for reflective X-ray optics, the majority of existing speckle-based methods fail to provide reliable 2D information about the optical surface being characterized. Compared with the 1D information typically output from speckled-based methods, a 2D map is more informative for understanding the overall quality of the optic being tested. In this paper, we propose a method for *in situ* 2D absolute metrology of weakly focusing X-ray mirrors. Importantly, the angular misalignment of the mirror can be easily corrected with the proposed 2D processing procedure. We hope the speckle pattern data processing method presented here will help to extend this technique to wider applications in the synchrotron radiation and X-ray free-electron laser communities.

## Abstract 2

This work extends the application of 2D speckle scanning techniques to the slope error measurement of weakly focusing reflective X-ray optics.

## 1. Introduction

With the development of the next generation of high-brightness X-ray sources, such as synchrotron radiation facilities and X-ray free-electron lasers (XFELs), increasingly high demands will be placed on fabricating and testing high-quality X-ray optical elements. Due to a variety of manufacturing limitations, currently available X-ray optics can present a number of different wavefront aberrations when used on synchrotron or XFEL sources. These aberrations will inevitably deteriorate the performance of X-ray optics at such facilities. *Ex situ* metrology techniques (Takacs *et al.*, 1989 ▸; Yamauchi *et al.*, 2003 ▸; Siewert *et al.*, 2004 ▸; Alcock *et al.*, 2016 ▸) are routinely used for the inspection of X-ray mirrors before they are installed on beamlines. These *ex situ* methods can provide valuable measurements of any inherent imperfections on the mirror surfaces. However, when the optics are installed on a beamline, the local mechanical, thermal and other environmental conditions can distort the mirrors further. As a result, *in situ* and at-wavelength measurements, under real working conditions, are becoming increasingly indispensable for providing detailed information about the performance of X-ray optics on synchrotron and XFEL sources.

Over the last few decades, many at-wavelength techniques (Hignette *et al.*, 1997 ▸; Yumoto *et al.*, 2006 ▸; Idir *et al.*, 2010 ▸; Kewish *et al.*, 2010 ▸; Bérujon, Wang, Ziegler & Sawhney, 2012 ▸; Sutter *et al.*, 2012 ▸; Assoufid *et al.*, 2016 ▸; Laundy & Sawhney, 2017 ▸; Moxham *et al.*, 2021 ▸) have been developed to characterize various types of X-ray optics. Among them, speckle-based methods (Wang *et al.*, 2015 ▸; Berujon *et al.*, 2014 ▸, 2020*a* ▸,*b* ▸; Zhou, Wang, Fox *et al.*, 2018 ▸; Wang, Kashyap & Sawhney, 2015*a* ▸) have become popular due to their relative simplicity in terms of experimental setup, relatively short data acquisition time and less stringent requirement for transverse coherence. Speckle-based at-wavelength metrology has been used for the measurement of X-ray reflecting mirrors (Wang *et al.*, 2015*b* ▸; Xue *et al.*, 2019 ▸); although, in these cases, either the influence of any wavefront error derived from the incident beam was ignored (Wang *et al.*, 2015*b* ▸) or a reliable 2D map of the wavefront slope error was not produced (Xue *et al.*, 2019 ▸). The wavefront error resulting from imperfections of upstream optics on a beamline can introduce significant systematic error for measurements of super-polished X-ray mirrors with slope errors <100 nrad. For example, it is essential to consider the wavefront error contribution from upstream optics and provide precise metrology information for the ion beam figuring (IBF, a form of corrective polishing) process (Siewert *et al.*, 2005 ▸; Idir *et al.*, 2015 ▸; Hand *et al.*, 2019 ▸).

The absolute measurement of X-ray optics, which separates the wavefront errors derived from the tested optics from those derived from upstream optics, is often desirable. Although absolute measurements of refractive optics have become routine (Berujon *et al.*, 2013 ▸; Wang, Kashyap & Sawhney, 2015a ▸), absolute measurements of reflective optics have only been proposed recently (Xue *et al.*, 2019 ▸). For X-ray refractive optics, the tested optics are usually assumed to be thin samples. However, this is not the case for X-ray reflective mirrors due to the small grazing-incidence angle. Only 1D slope errors of the X-ray mirror were retrieved in the previous work (Xue *et al.*, 2019 ▸), whereas here we extend the absolute measurement for X-ray reflective mirrors to the 2D case. The misalignment of the roll angle of the mirror can create artificial features in the 2D map of the wavefront slope error. Fortunately, this misalignment can be corrected using further data processing. In this paper, we describe an *in situ* at-wavelength absolute measurement of the slope error for a weakly focusing X-ray mirror and compare these results with *ex situ* measurements. We use the term weakly focusing to mean that the reflected beam is not strongly altered by the optic being tested relative to the incident X-ray beam. If the speckle patterns are present in both the direct beam and the reflected images, then those patterns are directly comparable. A flat mirror, a crystal or a single compound refractive lens element can all be classified as weakly focusing optics.

## 2. Principles of the 2D wavefront slope error measurement

Speckle phenomena are widely used for metrology and other experiments within the visible optics community (Goodman, 2007 ▸, 2015 ▸). In the last decade, speckle-based techniques have been extended to the X-ray region, both for X-ray imaging research (Bérujon, Ziegler, Cerbino & Peverini, 2012 ▸; Berujon, Wang & Sawhney, 2012 ▸; Morgan *et al.*, 2012 ▸; Zanette *et al.*, 2014 ▸; Berujon & Ziegler, 2015 ▸, 2016 ▸; Wang *et al.*, 2015*a* ▸, 2018 ▸; Wang, Kashyap & Sawhney, 2015*a* ▸,*b* ▸; Zhou *et al.*, 2015 ▸; Wang, Kashyap & Sawhney, 2016*a* ▸,*b* ▸; Zdora *et al.*, 2017 ▸) and for at-wavelength measurement of X-ray optics (Bérujon, Wang, Ziegler & Sawhney, 2012 ▸; Berujon *et al.*, 2014 ▸, 2020*a* ▸,*b* ▸; Wang, Kashyap & Sawhney, 2015*a* ▸, Wang *et al.*, 2015*b* ▸, Zhou, Wang, Fox *et al.*, 2018 ▸). A review of the use of speckle-based techniques in X-ray imaging has been published (Zdora, 2018 ▸). In the X-ray regime, speckle patterns can be generated by passing the radiation through various materials, such as sandpapers with different average grain sizes and cellulose acetate membranes (Kashyap *et al.*, 2015 ▸), and collecting an image with a detector placed downstream. The speckle grains act as probes of the wavefront propagation direction. When the speckle generator is translated across the beam, the speckle pattern on the detector plane will move accordingly. The detected displacement of the speckle pattern is derived from both the speckle generator movement and the wavefront propagation. By tracking the speckle pattern displacement on the detector plane, the local wavefront propagation direction can be recovered.

In general, the various speckle-based methods can be divided into two measurement modes: the differential mode (Wang, Kashyap & Sawhney, 2015*a* ▸; Xue *et al.*, 2019 ▸) and the self-reference mode (Berujon *et al.*, 2014 ▸; Wang *et al.*, 2015*b* ▸). For the self-reference mode, only one image stack is collected with the optic being tested placed in the X-ray beam while the speckle generator is scanned. The physical quantity obtained directly from this mode is the second derivative of the wavefront, which is analogous to the local curvature of the wavefront (Berujon *et al.*, 2014 ▸). Recent work has shown that the second derivative of the wavefront will severely impact the intensity variations in the far-field image (Hu *et al.*, 2021 ▸). The measured wavefront from the self-reference mode is a convolution of the incident beam wavefront with that derived from the optic being tested. The differential mode has been proposed to allow correction of the influence of the incident beam. Two image stacks, one with the optic being tested and another without the optic in the beam, are required in this mode. During data acquisition, the speckle generator is always present in the X-ray beam and the image stack measured without the optic being tested acts as a reference. The information directly obtained from the differential mode is the local wavefront slope error. In many cases, the reference beam is not always accessible at the detector plane, especially for strongly focusing optics where a large change in the field of view of the detector would be necessary.

The differential mode has been used since the early development of the X-ray speckle tracking (XST) technique (Bérujon, Ziegler, Cerbino & Peverini, 2012 ▸). This technique tracks only two images acquired by the detector, one from the reference beam and the other from the optic being tested. This method has limited spatial resolution in the scanned direction because a sub-region of the acquired image has to be chosen for accurate speckle pattern tracking. In order to obtain a higher spatial resolution for at-wavelength measurements, an X-ray speckle scanning (XSS) technique has been proposed (Berujon *et al.*, 2014 ▸). The XSS technique enables pixel-wise data analysis along the scan direction. In this paper, we make use of the differential-mode XSS technique.

**Figure 1:**
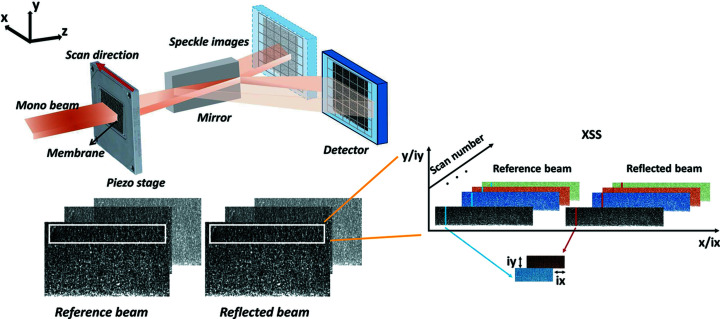
Experimental setup and data processing procedure for the 2D metrology of an X-ray mirror. The mirror was positioned to reflect horizontally to decouple the measurements from any vertical vibration and structure coming from the double-multilayer monochromator. The sandpaper speckle generator translated across the beam in the *x* direction. Two image stacks were acquired: (1) the reference beam image stack without the mirror being tested in the beam and (2) the reflected beam image stack with the mirror in the beam. Sub-regions of the raw images were selected for the 2D data analysis, as indicated by the white rectangles. In order to analyse the whole detecting area, the sub-regions were shifted to cover the full extent of the raw image. Column-by-column analysis was conducted in each sub-region. One column of pixels from each image in both image stacks was extracted and stitched together to form two new images, as shown on the right. The two new images were then used in a cross-correlation calculation to obtain the displacements in two directions, *iy* and *ix*. The 2D map of the displacements was generated by calculating the results for all of the columns within one sub-region and then shifting the sub-region to cover the whole raw image.

The experiment setup is illustrated in Fig. 1 ▸. The speckle pattern generator consisted of a sheet of sandpaper positioned upstream of the mirror being tested and mounted on a high-precision translation stage (Wang, Kashyap & Sawhney, 2015*a* ▸). The mirror was positioned to reflect horizontally to decouple the influence of the beam structure introduced by the double-multilayer monochromator and to maximize the stability of the mirror. For the smooth X-ray mirror, the critical direction of interest is along the mirror length. As shown in Fig. 1 ▸, translation of the speckle generator along the *x* direction is used to achieve the best possible spatial resolution along the length of the mirror. A single image was collected at each step of the speckle generator scan, producing a stack of images for the whole 1D translation. For the differential mode, two image stacks were obtained, one with the mirror being tested placed in the beam and one without the mirror. Both image stacks were taken as the speckle generator was scanned along the *x* direction.

A sub-region of the raw images, around 100 pixels along the mirror width (*y*) and the whole mirror length (*x*), was chosen for the column-by-column analysis, as shown in Fig. 1 ▸. The sub-regions (depicted by white rectangles in Fig. 1 ▸) were shifted during the data processing steps to cover the full extent of the collected images. The column-by-column analysis within the sub-regions was used to generate a 1D curve of the wavefront slope error. Every column of pixels (within the chosen sub-region) from each raw image in the stack was extracted and stitched together to form a new image, as shown on the right-hand side of Fig. 1 ▸. Two new images were thereby generated from the two image stacks and then cross-correlated to determine the speckle pattern displacements in two dimensions, *ix* and *iy*, with sub-pixel accuracy (Bing *et al.*, 2006 ▸). A 1D curve of the wavefront slope error was obtained by applying this procedure to all columns of pixels on the detector plane and the 2D map was formed by stitching the 1D curves from each of the sub-regions across the full raw images. If α_*x*_ and α_*y*_ represent the wavefront propagation direction at a certain position (*x*_0_, *y*_0_) on the detector plane, then the displacements *ix* and *iy* have the following relations

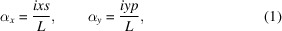

where *s* is the scan step size of the speckle generator, *p* is the pixel size of the detector, and *L* is the distance between the mirror centre and the detector plane. Note that equation (1) shows that the real speckle shift is in the units of the scan step size in the scan (*x*) direction, whereas it is in the units of the pixel size in the other (*y*) direction.

## 3. Experiment and results

A planar, silicon mirror with multiple coated lanes was measured using the differential-mode XSS technique. The experiment was conducted at the B16 Test beamline at the Diamond Light Source (Sawhney *et al.*, 2010 ▸). The X-ray radiation from the bending magnet source was passed through a double-multilayer monochromator (DMM) to select an energy of 15 keV before impinging on the mirror being tested. The DMM was employed to obtain a high incident beam flux that would improve the signal-to-noise ratio for a smaller detector pixel size. The mirror was placed to reflect horizontally to mitigate the effects of the horizontal striations derived from the upstream DMM and to achieve better stability. The mirror was 450 mm long and 60 mm wide. The lane tested on the mirror was coated with nickel, was 10 mm wide and the grazing angle was 3 mrad. A selection of commercially available sandpapers was used to generate the speckle pattern and was mounted on a 2D high-precision scanning piezo stage located ∼41 m from the bending magnet source. As shown in Fig. 1 ▸, the sandpapers were translated along the *x* direction with a scan step size of 1 µm. Images of the direct and reflected beam were recorded using an sCMOS camera (pco.edge) integrated with a Ce-doped LuAG scintillator attached to an optical lens system (Zhou, Wang, Connolley *et al.*, 2018 ▸). The detector was located ∼0.83 m downstream from the mirror centre and had an effective pixel size of 1.07 µm. The effective pixel size was chosen to obtain a better spatial resolution when the results were projected back to the mirror surface. In addition, each speckle grain on the image should cover several pixels on the detector. In theory, the smaller the scan step size of the speckle generator, the greater the sensitivity of the measurement. However, a smaller scan step size also requires a larger scan number and, hence, a longer scanning time. As a result, we chose a scan step of 1 µm as a compromise between sensitivity and data acquisition time.

### 3.1. Correction of mirror misalignment

**Figure 2:**
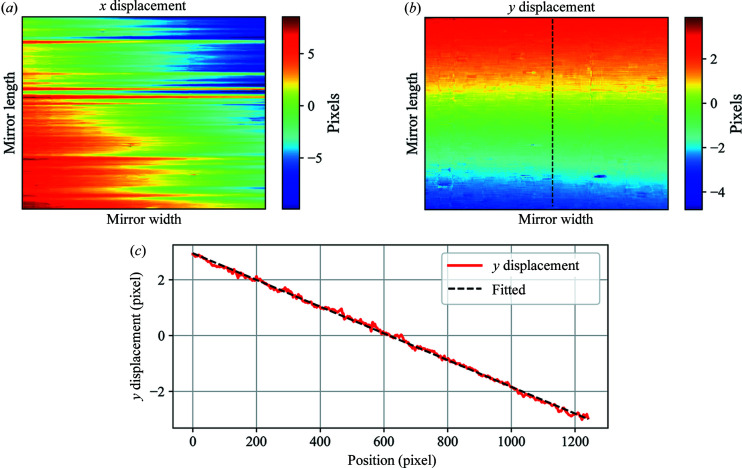
2D maps of the calculated displacements, without mirror misalignment correction, in the (*a*) *x* and (*b*) *y* directions. In the 2D maps the mirror width corresponds to the *y* direction and the mirror length corresponds to the *x* direction on the detector plane. The central line has been extracted from the 2D map of the *y* displacement (*b*) to give the 1D curve shown in (*c*). The *x* axis of (*c*) is the position along the mirror length.

When initially mounted on the beamline the mirror can be misaligned with respect to the incident X-ray beam. From equation (1) we know that to calculate the wavefront slope error we need to firstly calculate the speckle displacements *ix* and *iy*. Figs. 2 ▸(*a*) and 2(*b*) show the 2D maps of the calculated displacements in the two orthogonal directions without any correction for mirror misalignment. The mirror width corresponds to the *y* direction and the mirror length corresponds to the *x* direction on the detector plane. Thus, the *x* displacement *ix* was used to calculate the slope error along the mirror length. There are low-spatial-frequency variations in the 2D map for both *x* and *y* displacements. However, these variations are not generated by any real mirror surface error. Mirrors commonly used as X-ray optics are typically very smooth and approximately uniform along their width. Thus, if a mirror reflects horizontally, as in this case, then the speckle pattern displacements in the *y* direction on the detector plane should not change significantly.

**Figure 3:**
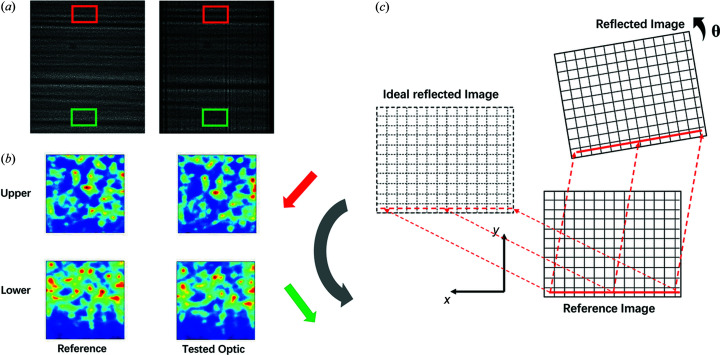
(*a*) Raw speckle images of the reference beam (left) and the reflected beam (right) from the optic being tested. The magnified speckle patterns from the sub-regions (red and green rectangular boxes), extracted from (*a*) are shown in (*b*). The red arrow shows the shift of the speckle pattern from the upper part of the image of the reference beam to the upper part of image of the reflected beam. Likewise, the green arrow shows the shift of the speckle pattern extracted from the lower parts of the reference and reflected images. Comparing the speckle pattern shifts from the upper part and the lower part of the two raw images together, we observe that the image of the reflected beam is rotated with respect to the image of the reference beam. Panel (*c*) demonstrates how the rotation angle affects the calculated *y* displacement. The *y* direction is along the mirror width. Since the optical surface of the X-ray mirror should be very smooth, the calculated *y* displacement *iy* should be small along the mirror length while the speckle generator is translated along the *x* direction. If the mirror is well aligned, the calculated *iy* should be close to zero. When the mirror is rotated about the *z* axis (not shown in the image), the reflected image will be rotated by the same angle. As shown in (*c*), the calculated *iy* will have a linear relationship with the *x* coordinates.

By comparing the upper and lower sub-regions in the raw speckle images of the reference beam and the reflected beam in detail [as shown in Figs. 3 ▸(*a*) and 3(*b*)], we observed that the raw images in the two image stacks are slightly rotated. The rotation angle can affect the 2D map of the *y* displacement, as shown in Fig. 3 ▸(*c*). When the speckle generator was scanned along the *x* direction perpendicular to the incident beam, the calculated displacement in the vertical direction *iy* should be small if the mirror is well aligned because of the very smooth surface. The linear variation in *iy*, shown in Fig. 2 ▸(*c*), can therefore be attributed to rotation of the mirror with respect to the incident beam. The rotation angle can be recovered from the gradient of the plot of *iy* against the pixel position. In this case, the reflected images were rotated by 0.275° relative to the reference images. As well as the rotational misalignment of the mirror measured here, a twist error for the mirror will also be convoluted in the results. This mirror twist effect was much more difficult to separate from the main rotational component for this at-wavelength experiment. Unlike the rotation of the mirror relative to the reference beam, the twist of the mirror can be regarded as a form of surface error. After correction of the rotational misalignment, the measured 2D maps of the *x* and *y* displacements reveal the required surface information for the mirror.

**Figure 4:**
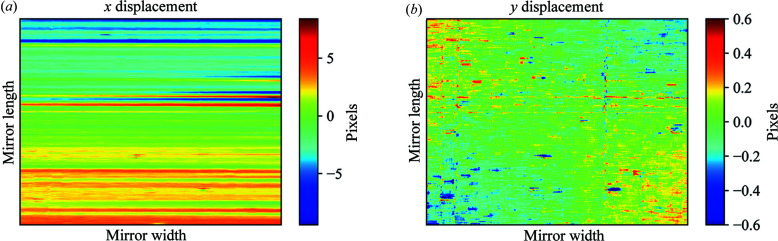
Rotation-corrected 2D map of the displacement in the (*a*) *x* and (*b*) *y* directions. The *y* displacement map (*b*) has none of the large-scale artificial low-spatial-frequency features derived from the rotational misalignment observed in Fig. 2 ▸(*b*). The *x* displacement was used to calculate the wavefront slope error.

Figs. 4 ▸(*a*) and 4(*b*) show the rotation-corrected 2D maps of the calculated displacements in the *x* and *y* directions. Comparing the post-correction maps (Fig. 4 ▸) with the pre-correction maps (Fig. 2 ▸), it is clear that the low-spatial-frequency features have disappeared.

We have demonstrated that the misalignment of the mirror relative to the incident beam is commonplace, irrespective of how accurately the mirror being tested is mounted mechanically beforehand. The high sensitivity of the speckle-based technique allows this misalignment to be corrected from data processing.

### 3.2. Results with different incident beam wavefronts

**Figure 5:**
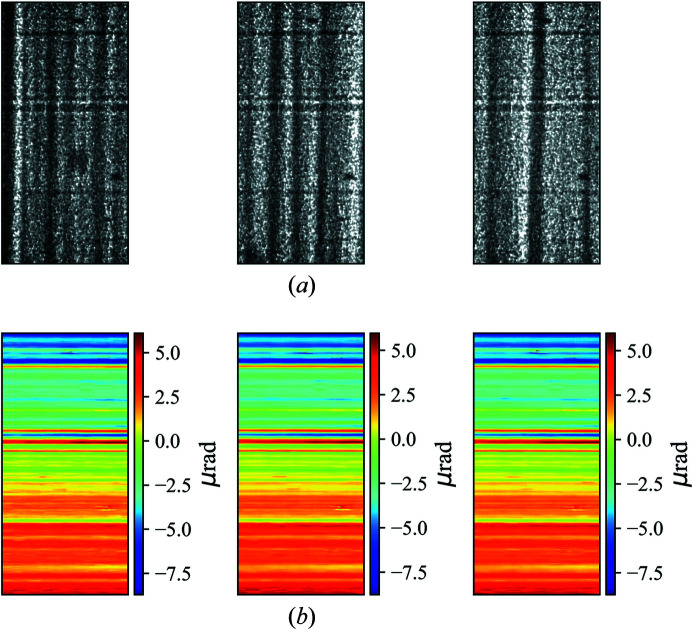
(*a*) Raw speckle images at three different positions of the DMM. These images have been rotated by 90°. The pattern of vertical stripes from the multilayer structure of the monochromator varies between each position providing three different incident wavefronts. (*b*) The corresponding calculated 2D maps of the wavefront slope error showing no obvious variation between them. The horizontally reflecting setup of the mirror being tested decouples the strong vertical variations derived from the DMM.

To estimate how the wavefront of the incident beam influences the speckle-based measurements of the optic being tested, the DMM assembly upstream of the mirror was translated horizontally. This provided an incident beam wavefront derived from different areas of the monochromator, as shown in Fig. 5 ▸(*a*). The images collected all show a background of striations created by the multilayer structure of the DMM. The mirror being tested was exposed to three different parts of the monochromator and, therefore, different incident wavefronts. Despite this, the calculated wavefront slope errors at the detector plane for these three cases were almost identical, as shown in Fig. 5 ▸(*b*). The variation in the standard deviation for the three 2D maps of the wavefront slope error was less than 0.5%. This result shows that mounting the mirror on its side to reflect horizontally can effectively decouple the strong vertical variations that are present in the incident wavefront from the DMM.

**Figure 6:**
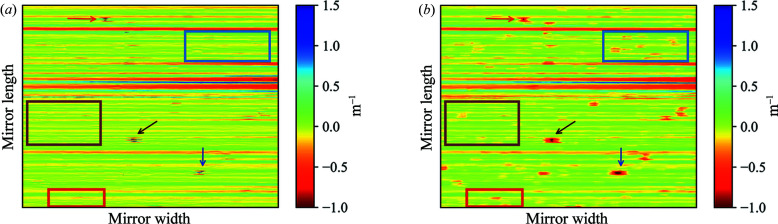
(*a*) The 2D map of the wavefront slope error measured using the differential-mode XSS technique was differentiated to produce a 2D map of the incident-beam-corrected wavefront curvature error shown here. (*b*) 2D map of the wavefront curvature error from the self-reference XSS technique for comparison. The errors derived from the incident beam wavefront can be easily observed in (*b*). The rectangular boxes demarcate areas in which artefacts in the wavefront of the incident beam are substantially reduced when using the differential-mode XSS technique. The arrows indicate strong artefacts that are not completely removed.

Next, we compared the 2D results from our incident-beam-corrected differential-mode XSS method with those from the self-reference XSS technique (Berujon *et al.*, 2014 ▸; Wang *et al.*, 2015*b* ▸), which were collected without a reference image stack. To allow a direct comparison of the two techniques, we differentiated the 2D map of the wavefront slope error obtained using the differential-mode XSS technique along the mirror length to obtain a 2D map of the wavefront curvature error. A comparison of these two wavefront curvature error maps is made in Fig. 6 ▸. The rectangular boxes mark out the areas in which artefacts in the wavefront of the incident beam are substantially reduced when using the differential-mode XSS technique employed in this paper. Strong artefacts in the incident beam wavefront can only be partially reduced using this technique.

### 3.3. Mirror surface slope error

**Figure 7:**
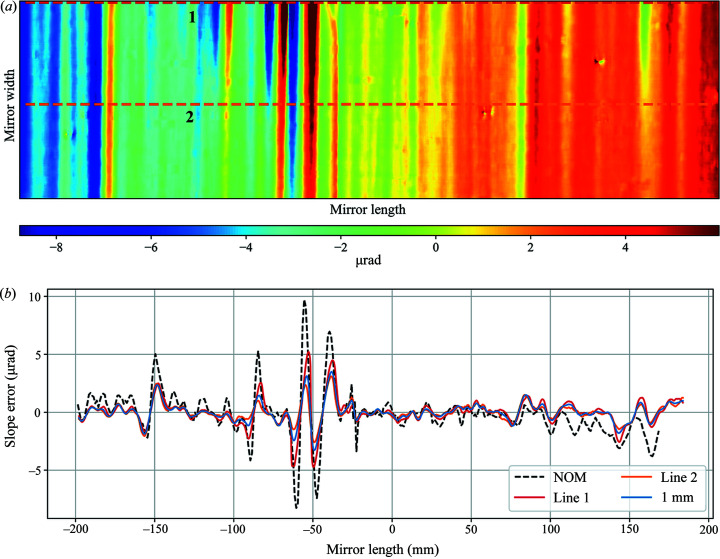
(*a*) Calculated wavefront slope error using the differential-mode XSS technique. Slope errors along the dashed orange line (centre) and red line (upper edge) were extracted for comparion with the Diamond-NOM metrology results. An area of ∼1 mm width (half the image) from the 2D map of the wavefront slope error was extracted to match the size of the probe used for the *ex situ* measurement. The calculated slope errors within this area were averaged along the mirror width. (*b*) Slope errors projected back to the mirror plane. The Diamond-NOM result is shown as a dashed black line. The red and orange lines correspond to the upper edge and the centre of the measured area, respectively. The blue line is the averaged slope error corresponding to a 1 mm mirror width.

To compare the at-wavelength results with the visible light metrology results, the X-ray wavefront measured at the detector plane needs to be projected back to the mirror surface plane. This process leads to additional complications due to the length of the mirror which, unlike compound refractive lenses (Wang, Kashyap & Sawhney, 2015*a* ▸), cannot be treated as a thin optic. Fig. 7 ▸(*a*) shows the 2D map of the wavefront slope error covering the full height of the detector field of view which corresponds to 1.68 mm of the width of the mirror. For a planar mirror, to determine the mirror coordinates it is possible to project the detector plane coordinates back to the mirror plane using a linear relationship if the grazing angle and the distance between the mirror and the detector plane are known. A more universal and self-consistent method is to use an iterative algorithm proposed in early works (Berujon & Ziegler, 2012 ▸; Berujon *et al.*, 2014 ▸). Here, we show the results obtained using this universal iterative algorithm.

Fig. 7 ▸(*b*) compares *ex situ* measurements of the same mirror made in the Optics Metrology Lab at the Diamond Light Source using the Diamond-NOM (Nanometre Optical Metrology) apparatus (Alcock *et al.*, 2010 ▸, 2016 ▸) and the at-wavelength speckle-based measurements made on the B16 beamline. The two 1D slope error measurements were extracted from the edge (line 1) and centre (line 2) of the imaged area of the mirror, as shown in Fig. 7 ▸(*a*). The line profiles can be fitted with a linear polynomial which corresponds to a cylindrical shape error. There is good agreement between the features in the *ex situ* and *in situ* measurements of the mirror shown in Fig. 7 ▸(*b*). Due to the hardware limitations, the area of the mirror being tested probed by the *ex situ* measurement on the Diamond-NOM and the *in situ* at-wavelength measurement did not overlap exactly. This can explain the disparities from some amplitudes of the features in the slope error curves. It is also likely that the speckle tracking technique is less accurate for sharp or pronounced changes in the slope error.

Various experimental setups, such as placing the speckle generator upstream or downstream of the optic, scanning the speckle generator one-dimensionally, diagonally or in a spiral, can also be used to measure the X-ray reflective optics. Here we have demonstrated the necessity of correcting the rotation of the optic being tested relative to the reference beam. The rotation derived from misalignment will create artificial large-scale features in the 2D map of the wavefront slope error. Owing to the small grazing angle inherent with X-ray reflective optics, the spatial resolution along the mirror length varies from around 0.1 mm to several millimetres. The resolution is determined by the detector pixel size projected back to the mirror surface. For a 1 µm pixel size at the detector and a 3 mrad grazing angle, the achievable spatial resolution at the mirror would be 1 µm/3 mrad ≃ 0.33 mm.

The differential-mode XSS technique is not limited to measurement of the weakly focusing mirrors but can also find application in the characterization of deformable mirrors (Wang *et al.*, 2015 ▸; Sutter *et al.*, 2016 ▸). The method can be used to determine the slope piezo response functions of the mirror if the wavefront of the free state of the mirror is used as a reference beam.

## 4. Conclusions

We applied the differential-mode XSS technique to characterize a weakly focusing X-ray reflecting mirror. We obtained 2D maps for the mirror wavefront slope error and showed that the misalignment of the mirror with respect to the incident beam can be corrected using the acquired speckle-based images. By positioning the mirror to reflect the beam horizontally, the influence of the striations from the upstream monochromator optics were successfully decoupled from the slope error measurement. We have also shown that errors in the wavefront from the incident beam can be minimized using the differential-mode XSS method. At-wavelength measurements for the X-ray mirror show good agreement with the *ex situ* metrology which used visible light.

The differential-mode XSS technique has been used widely in previous work for measuring different types of X-ray optics, including the characterization of thin X-ray optics such as compound refractive lenses. We have extended the differential-mode XSS technique to the measurement of X-ray reflective optics. We hope that our proposed data processing method can extend the XSS technique to *in situ* slope error measurements of weakly focusing X-ray reflective optics.
